# Factors Affecting Arterial Stiffness and Brachial-Ankle Pulse Wave Velocity in Patients With Type 2 Diabetes

**DOI:** 10.7759/cureus.68561

**Published:** 2024-09-03

**Authors:** Tomoki Furuya, Shinji Kitahama, Yuma Tamura, Susumu Ogawa, Yuki Nakatani, Takanori Yasu

**Affiliations:** 1 Department of Cardiovascular Medicine and Nephrology, Dokkyo Medical University Nikko Medical Center, Nikko, JPN; 2 Research Team for Social Participation and Healthy Aging, Tokyo Metropolitan Institute for Geriatrics and Gerontology, Itabashi, JPN; 3 Department of Physical Therapy, Igaku Academy, Kawagoe, JPN; 4 Department of Endocrinology, Kawatsuru Plaza Clinic, Kawagoe, JPN; 5 Department of Rehabilitation, Dokkyo Medical University Nikko Medical Center, Nikko, JPN; 6 Department of Diabetes and Endocrinology, Dokkyo Medical University Nikko Medical Center, Nikko, JPN

**Keywords:** atherosclerosis, sarcopenia, type 2 diabetes, metabolic paradox, body mass index

## Abstract

Background

We defined the progression of atherosclerosis in patients by brachial-ankle pulse wave velocity (ba-PWV) and determined the relationship between ba-PWV and body composition factors, muscle strength, and patient characteristics.

Methodology

The arterial stiffness index, body composition, anthropometric parameters, blood test data, and background factors were evaluated in 109 patients with type 2 diabetes mellitus (T2DM). Statistical analysis was conducted using logistic regression analysis and analysis of covariance (p < 0.05).

Results

The mean age of the participants was 62.87 ± 12.11 years, body mass index (BMI) was 25.72 ± 4.35 kg/m², ba-PWV was 1653.08 ± 366.55 cm/s, systolic blood pressure was 138.87 ± 16.74 mmHg, the number of years of disease was 11.17 ± 9.51 years, and hemoglobin A1c value was 6.90 ± 0.74%. Binomial logistic regression analysis of ba-PWV divided into two groups by arterial stiffness index, ≥ (or <) 1,400 cm/s, showed systolic blood pressure (odds ratio = 1.11, 95% confidence interval = 1.05-1.18, p < 0.001), BMI, and number of years of disease were significant independent variables. The cut-off value for BMI was 26.28 kg/m². BMI was a significant explanatory factor for ba-PWV in the analysis of covariance (p < 0.001).

Conclusion

BMI was associated with the incidence of atherosclerosis in patients with T2DM. We proposed a cut-off value for BMI below which the atherosclerosis index increased, a result that may reflect the influence of the metabolic paradox.

## Introduction

Type 2 diabetes mellitus (T2DM) causes vascular endothelial dysfunction. Many patients present with hypertension. Clinically, T2DM is associated with atherosclerosis characterized by young onset and progression [1]. The duration of T2DM is independently associated with brachial-ankle pulse wave velocity (ba-PWV), even after adjusting for age, blood pressure, heart rate, cardiovascular events, and metabolic syndrome [2]. Furthermore, ba-PWV in patients with mild T2DM (hemoglobin A1c (HbA1c) = 6.6%), including those without T2DM (HbA1c = 5.8%), increased with the blood glucose level and showed a significant positive correlation with the waist circumference and waist-to-hip ratio [3]. Furthermore, the progression of arterial stiffness measured by ba-PWV predicts the risk of all-cause and cardiovascular mortality in T2DM, and the prognostic value of ba-PWV has been reported [4]. These results suggest that the duration of T2DM, body composition, and risk of event occurrence are closely related to atherosclerosis.

It is essential to prevent the development and progression of diabetic microvascular complications and atherosclerotic diseases while treating patients with T2DM [5]. Therefore, medical professionals who interact with patients with T2DM daily need to confirm the presence or absence of complications and evaluate the presence or progression of atherosclerosis. In addition, considering that T2DM is a chronic metabolic disease and that most patients are treated as outpatients [6], most situations where medical professionals are involved in treating such patients are expected to be at home or in small medical facilities, such as clinics.

ba-PWV is an important index that reflects the elastic properties of arteries. Arteries have a three-layered structure of intima, tunica media, and adventitia, and pressure waves associated with blood flow propagate through these layers. As the arterial wall stiffens, the velocity of pressure wave propagation increases. ba-PWV is a method for quantitatively evaluating the progression of arterial stiffness and the elastic properties of arteries by measuring the pulse wave velocity between the upper arm and ankle and is useful in the early diagnosis and prognosis of arteriosclerosis. Generally, ba-PWV is measured by specialized instruments. However, methods to assess arterial stiffness are limited [7].

It is important to evaluate arterial stiffness to achieve treatment goals in patients with T2DM. Therefore, this study aimed to evaluate body composition, muscle strength, and patient characteristics in patients with T2DM and to identify factors that influence the progression of arterial stiffness.

## Materials and methods

**Figure 1:**
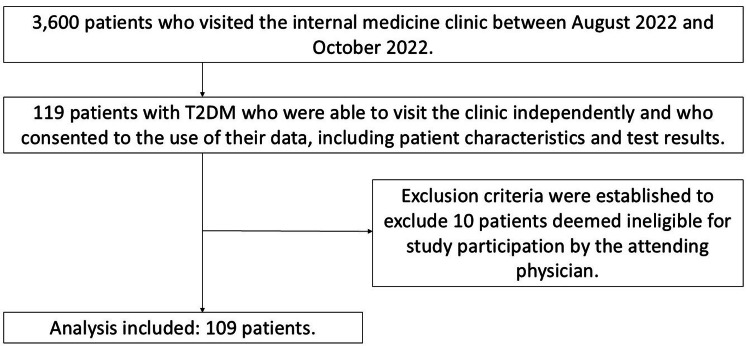
Procedures for inclusion and exclusion of subjects. A total of 119 patients with T2DM who could be seen independently from 1100 patients who visited the internal medicine clinic between August 2022 and October 2022 and who consented to the use of their data, including patient characteristics and test results, were included. Ten patients who met the exclusion criteria were excluded, and 109 patients were included in the analysis. T2DM: type 2 diabetes mellitus.

This study is a prospective observational study. The study population consisted of 3,600 patients who visited the internal medicine clinic from August 2022 to October 2022. Overall, 119 patients with T2DM who were able to visit the hospital independently and who gave consent for the use of data on patient characteristics and test results were included in the study. The exclusion criteria were type 1 diabetes, lower extremity movement disorder (not excluded in the absence of edema/swelling of the lower extremities), limb defects, apparent paralysis due to central nervous system disorder, an ankle-brachial index (ABI) value of ≤0.9, suspected lower extremity arterial occlusion, malignant neoplasm, pregnancy or suspected pregnancy, cardiac pacemaker or other implants in the body, nonpitting edema, and severe lower extremity edema (Fukasawa variant) [8]. We also excluded other patients deemed ineligible for participation in the study by the attending physician. In total, 10 patients were excluded based on the aforementioned criteria, and 109 were included in the analysis (Figure 1).

This study was designed in accordance with the principles of the Declaration of Helsinki and ethical guidelines for life science and medical research involving human subjects. It was conducted with the approval of the Ethics Committee of Dokkyo Medical University (approval number: Nikko 30015; dated: April 16, 2019). Written informed consent was obtained from all study participants.

Body composition was assessed by measuring the lower limb circumference and bioelectrical impedance analysis (BIA). BIA was performed by a body composition analyzer (DC-430A-P, TANITA, Tokyo, Japan) to evaluate skeletal muscle mass and fat mass. Skeletal muscle mass and fat mass were divided by the body weight to obtain the muscle-to-weight ratio and body fat percentage, respectively. The body mass index (BMI) was calculated from height and weight measurements [9].

Muscle strength indices were measured by a hand-held dynamometer (μ-tas MT1, Anima Corporation, Tokyo, Japan) to measure knee extension muscle strength and ankle plantar flexion muscle strength in the sitting position. Knee extensor strength and ankle plantar flexor strength were calculated as the product of the value obtained by the hand-held dynamometer (kgf) and lower leg length (m) and corrected by dividing by muscle mass (kg) and body weight (kg), respectively [10]. Grip strength was measured twice with the dominant hand by a standard digital grip strength meter (Grip-D, Takei Scientific Instruments Co., Tokyo, Japan). The handle of the grip strength meter was adjusted to the height of the proximal interphalangeal joint before measurement. The obtained values were divided by the body weight to calculate the grip strength-to-weight ratio.

The ABI value, systolic blood pressure (SBP) (mmHg), and ba-PWV (cm/s) were measured as indices of arterial stiffness. These were measured by a blood pressure pulse wave measuring device (HBP-8000, Fukuda Colin Co., Ltd., Tokyo, Japan) after resting in the supine position for at least 10 minutes in an air-conditioned room (approximately 26°C).

Clinical diabetic neuropathy was assessed by two prerequisites and three neurological examination items. The prerequisites were (1) a diagnosis of diabetes mellitus and (2) the ability to exclude neurological diseases other than diabetic peripheral neuropathy (DPN). DPN was defined as the presence of two or more of the following criteria: (1) subjective symptoms that may be caused by DPN; (2) decreased bilateral endocardial vibration thresholds; and (3) abnormal bilateral Achilles tendon reflexes [11]. Vibration thresholds of the bilateral endocardium were measured in the supine position by a tuning fork. The Achilles tendon reflex was measured in the supine position by a percussion instrument.

Blood laboratory data, including the blood glucose level (mg/dL), triglyceride level (mg/dL), and HbA1c (%) at any time, were collected from the medical records. These data were collected within two months of the date of other evaluations. The number of years of T2DM was calculated from the date of diagnosis of T2DM, which was collected from the medical records.

Statistical analyses were performed by comparing the difference in each parameter between those with ba-PWV above and below the age-specific reference value by the uncorrelated t and χ2 tests. Age-specific reference values for ba-PWV were calculated by the regression equation (Y=13.68X+678.82 for male individuals and Y=15.95X+514.69 for female individuals), as reported by Kobayashi et al. [12].

The correlation between ba-PWV and each test value was examined by Pearson’s product ratio correlation coefficient test for parametric data and Spearman’s rank correlation coefficient test for nonparametric data.

Multiple regression analysis (stepwise variable selection) with ba-PWV as the dependent variable was used to examine factors affecting ba-PWV. The independent variables included the number of years with T2DM (years) [13], negative Achilles tendon reflex [14], knee extensor muscle strength (kgf-m/kg/body weight) [15], BMI (kg/m2) [16], HbA1c (%) [17], and sex [18] as factors that have shown association in previous studies. These factors were adjusted for age (years) [13,16,17] and SBP (mmHg) [13,16,17].

Binomial logistic regression analysis (stepwise variable selection) was performed to investigate the effect of each test value on arterial stiffness. The dependent variable was defined as 1 (ba-PWV > 1,400 cm/s) for the presence of arterial stiffness and 0 (ba-PWV < 1,400 cm/s) for the absence of arterial stiffness, based on previous studies [19].

The cut-off values for BMI for arterial stiffness were calculated by receiver operating characteristic curves. The positive rate, true prevalence, sensitivity, specificity, positive predictive value, negative predictive value, positive likelihood ratio, and negative likelihood ratio were calculated to evaluate the accuracy of the obtained cut-off values.

To examine the relationship between ba-PWV and BMI, considering the effect of aging, we conducted an analysis of covariance (ANCOVA) with ba-PWV as the dependent variable and age as the independent variable, grouping participants according to the cut-off BMI value. All variables were preliminarily checked for normality by Q-Q plots and the Shapiro-Wilk normality test. Statistical analyses were performed by the data analyzed with IBM SPSS v.23 (IBM Corp., Armonk, NY, USA). The significance level was set at p < 0.05.

## Results

Among the 3,600 people who visited the clinic in question during the study period, of 119 participants who gave informed consent, 109 were selected for analysis in accordance with the exclusion criteria. Table 1 shows the main characteristics of the participants included in the analysis.

**Table 1:** Main characteristics of the sample studied (n = 109). No correspondence t-test, χ2 test, p < 0.05. ABI: ankle-brachial index; ACE-I: angiotensin-converting enzyme inhibitor; ARB: angiotensin receptor blocker; ASO: arteriosclerosis obliterans; ba-PWV: brachial-ankle pulse wave velocity; BMI: body mass index; CCB: calcium channel blocker; CKD: chronic kidney disease; COPD: chronic obstructive pulmonary disease; DPP-4: dipeptidyl peptidase-4; α-GI: α-glucosidase inhibitor; GLP-1: glucagon-like peptide-1; HbA1c: hemoglobin A1c; HDL-cho: high-density lipoprotein cholesterol; HT: hypertension; SBP: systolic blood pressure; SGLT-2: sodium-glucose cotransporter-2; SU: sulfonylurea.

			Group by ba-PWV		
		All participants	Participants below the standard value (ba-PWV < 1,400 cm/s)	Participants above the standard value (ba-PWV > 1,400 cm/s)	p-value
Variable	Unit	n = 109	n = 40	n = 69	
Number of male individuals	Person (%)	67 (61.5)	25 (62.5)	42 (60.9)	1
Age	years (SD)	62.87 (12.11)	61.30 (11.84)	63.78 (12.26)	0.305
BMI	kg/m^2^ (SD)	25.72 (4.35)	26.76 (4.69)	25.12 (4.05)	0.057
Body weight	kg (SD)	67.92 (15.01)	71.66 (16.07)	65.74 (14.03)	<0.05
Muscle mass	kg (SD)	44.80 (9.35)	46.19 (9.91)	44.00 (8.99)	0.24
Fatty constitution	kg (SD)	20.86 (8.94)	23.63 (10.16)	19.25 (7.78)	<0.05
Ankle extensor strength	kgf・m/kg/body weight (SD)	22.41 (10.86)	20.94 (8.55)	23.27 (11.97)	0.282
Knee joint extensor strength	kgf・m/kg/body weight (SD)	14.01 (4.21)	14.57 (4.48)	13.68 (4.04)	0.288
Grip strength-to-weight ratio	kg/body weight (SD)	0.44 (0.13)	0.43 (0.12)	0.46 (0.13)	0.237
ba-PWV	cm/s (SD)	1653.08 (366.55)	1347.97 (145.69)	1829.96 (338.70)	<0.001
ba-PWV, ≥1,400 cm/s (with arteriosclerosis)	Person (%)	69 (63.3)	19 (46.3)	50 (-73.5)	<0.01
ABI	‐	1.12 (0.08)	1.10 (0.10)	1.13 (0.07)	0.171
SBP	mmHg (SD)	138.87 (16.74)	131.57 (14.75)	143.10 (16.45)	<0.001
Achilles tendon reflex negative	(%)	52 (47.7)	15 (37.5)	37 (53.6)	0.116
Subjective symptoms present	(%)	17 (15.6)	7 (17.5)	10 (14.5)	0.785
Peripheral neuropathy present	(%)	69 (63.3)	28 (70.0)	41 (59.4)	0.307
Impaired vibration perception	(%)	72 (66.1)	26 (65.0)	46 (66.7)	1
Number of peripheral neuropathy symptoms	Pieces (SD)	1.29 (0.99)	1.20 (0.94)	1.35 (1.03)	0.457
Number of years of disease	Years (SD)	11.17 (9.51)	9.47 (9.28)	12.16 (9.56)	0.156
HbA1c	% (SD)	6.90 (0.74)	6.70 (0.69)	7.01 (0.74)	<0.05
Blood glucose level at any time	mg/dL (SD)	152.85 (52.60)	138.43 (38.30)	161.22 (57.96)	<0.05
Triglyceride	mg/dL (SD)	160.92 (122.22)	143.05 (74.15)	171.28 (142.38)	0.247
HDL-cho	mg/dL (SD)	57.37 (14.46)	57.60 (17.77)	57.23 (12.30)	0.899
ASO	Person (%)	1 (0.9)	0 (0.0)	1 (1.4)	1
COPD	Person (%)	1 (0.9)	0 (0.0)	1 (1.4)	1
Hepatitis C virus	Person (%)	1 (0.9)	0 (0.0)	1 (1.4)	1
HT	Person (%)	77 (70.6)	29 (72.5)	48 (69.6)	0.829
Graves’ disease	Person (%)	4 (3.7)	2 (5.0)	2 (2.9)	0.623
Ischemic heart disease	Person (%)	10 (9.2)	2 (5.0)	8 (11.6)	0.32
Ischemic cerebrovascular disease	Person (%)	8 (7.3)	2 (5.0)	6 (8.7)	0.708
Hashimoto’s thyroiditis	Person (%)	2 (1.8)	1 (2.5)	1 (1.4)	1
Hypothyroidism	Person (%)	4 (3.7)	2 (5.0)	2 (2.9)	0.623
Hypercholesterolemia	Person (%)	59 (54.1)	20 (50.0)	39 (56.5)	0.554
Hyperlipidemia	Person (%)	67.92 (15.01)	6 (15.0)	9 (13.0)	0.78
Hyperuricemia	Person (%)	100 (91.7)	6 (15.0)	3 (4.3)	0.072
Osteoporosis	Person (%)	4 (3.7)	3 (7.5)	1 (1.4)	0.139
Fatty liver	Person (%)	1 (0.9)	0 (0.0)	1 (1.4)	1
Chronic hepatitis	Person (%)	12 (11.0)	3 (7.5)	9 (13.0)	0.53
Chronic cardiac insufficiency	Person (%)	1 (0.9)	0 (0.0)	1 (1.4)	1
CKD	Person (%)	1 (0.9)	0 (0.0)	1 (1.4)	1
ACE-I	Person (%)	3 (2.75)	2 (5.00)	1 (1.45)	‐
ARB	Person (%)	53 (48.62)	24 (60.00)	29 (42.03)	‐
CCB	Person (%)	53 (48.62)	19 (47.50)	34 (49.28)	‐
DPP-4	Person (%)	57 (52.29)	17 (42.50)	40 (57.97)	‐
GLP-1	Person (%)	11 (10.09)	3 (7.50)	8 (11.59)	‐
SGLT-2	Person (%)	25 (22.96)	11 (27.5)	14 (20.29)	‐
α-GI	Person (%)	51 (46.79)	18 (45.00)	33 (47.83)	‐
Insulin	Person (%)	20 (18.35)	5 (12.50)	15 (21.74)	‐
Hyperlipidemic drugs other than statins	Person (%)	18 (16.51)	6 (15.00)	12 (17.39)	‐
Statin	Person (%)	59 (54.13)	24 (60.00)	35 (50.72)	‐
SU	Person (%)	6 (5.50)	2 (5.00)	4 (5.80)	‐
Other antihypertensive drugs	Person (%)	14 (12.84)	7 (17.50)	7 (10.14)	‐
Biguanide (metformin)	Person (%)	38 (34.86)	14 (35.00)	24 (34.78)	‐
Rapid-acting insulin secretagogue (glinide)	Person (%)	8 (7.34)	1 (2.50)	7 (10.145)	‐

The mean number of years of diabetes mellitus among the participants was 11.17 ± 9.51 years, and over 50％ of the patients had a normal weight (BMI = 18.5-24.9 kg/m2). Table 2 shows the relationship between the participants’ years of diabetes mellitus and BMI.

**Table 2:** Relationship between years of diabetes mellitus and BMI. The number of years affected by “thinness” is expressed as a mean value (minimum-maximum), considering the number of samples. SD: standard deviation; BMI: body mass index.

Physique	Leptosomatic habit, BMI >18.5 kg/m^2^	Normal, BMI = 18.5-24.9 kg/m^2^	Being overweight by one degree, BMI = 25.0-29.9 kg/m^2^	Being overweight by two degrees, BMI = 30.0-34.9 kg/m^2^	Being overweight by three degrees, BMI = 35.0-40.0 kg/m^2^
n (%)	2 (1.80)	52 (47.78)	38 (34.88)	12 (11.00)	5 (4.58)
Number of years of illness, years (SD)	4.00 (17.82-18.46)	13.10 (10.92)	10.08 (7.96)	10.08 (8.27)	5.00 (2.92)
BMI, kg/m^2^ (SD)	18.14 (0.46)	22.53 (1.87)	27.06 (1.24)	32.18 (1.54)	36.32 (0.66)

Correlations were found between ba-PWV and age, ba-PWV (>1,400 cm/s, with arterial stiffness), ABI value, blood pressure, muscle mass, fat mass, knee joint muscle strength, BMI, weight, number of symptoms of peripheral neuropathy, years of disease, blood sugar at any time, and ischemic cerebrovascular disease (Table 3).

**Table 3:** Correlation between ba-PWV and each parameter. Pearson and Spearman, p < 0.05. ABI: ankle-brachial index; ba-PWV: brachial-ankle pulse wave velocity; SBP: systolic blood pressure; BMI: body mass index; HbA1c: hemoglobin A1C; HDL-cho: high-density lipoprotein cholesterol; ASO: arteriosclerosis obliterans; COPD: chronic obstructive pulmonary disease; HT: hypertension; CKD: chronic kidney disease; SD: standard deviation.

Variable	Unit	r (ρ)	p-value
Sex	Person (%)	-0.16	0.099
Age	Person (%)	0.57	0.001
ba-PWV, ≥1,400 cm/s (with arteriosclerosis)	Person (%)	0.78	0.001
ABI	‐	0.24	0.05
SBP	mmHg (SD)	0.54	0.001
Muscle mass	kg (SD)	-0.35	0.001
Fatty constitution	kg (SD)	-0.40	0.001
Ankle extensor strength	kgf・m/kg/body weight (SD)	-0.06	0.566
Knee joint extensor strength	kgf・m/kg/body weight (SD)	-0.22	0.05
Grip strength-to-weight ratio	kg/body weight (SD)	-0.01	0.946
BMI	kg/m^2^ (SD)	-0.42	0.001
Body weight	kg (SD)	-0.46	0.001
Triglyceride	mg/dL (SD)	-0.06	0.567
Achilles tendon reflex negative	(%)	0.18	0.064
Subjective symptoms present	(%)	-0.05	0.592
Peripheral neuropathy present	(%)	0.08	0.392
Impaired vibration perception	(%)	0.14	0.142
Number of peripheral neuropathy symptoms	Pieces (SD)	0.20	0.05
Number of years of disease	Years (SD)	0.19	0.05
HbA1c	% (SD)	0.18	0.066
Blood glucose level at any time	mg/dL (SD)	0.32	0.001
HDL-cho	mg/dL (SD)	0.08	0.409
ASO	Person (%)	0.08	0.411
COPD	Person (%)	0.07	0.467
Hepatitis C virus	Person (%)	0.07	0.467
HT	Person (%)	0.17	0.081
Graves’ disease	Person (%)	0.00	0.962
Ischemic heart disease	Person (%)	0.14	0.144
Ischemic cerebrovascular disease	Person (%)	0.23	0.05
Hashimoto’s thyroiditis	Person (%)	-0.03	0.754
Hypothyroidism	Person (%)	-0.02	0.798
Hypercholesterolemia	Person (%)	0.15	0.114
Hyperlipidemia	Person (%)	-0.05	0.615
Hyperuricemia	Person (%)	-0.10	0.303
Osteoporosis	Person (%)	-0.07	0.501
Fatty liver	Person (%)	-0.01	0.925
Chronic hepatitis	Person (%)	-0.03	0.733
Chronic cardiac insufficiency	Person (%)	0.12	0.211
CKD	Person (%)	0.16	0.092

After adjusting for age and blood pressure, the association between ba-PWV and sex, blood pressure, BMI, knee extensor strength, number of years affected, negative Achilles tendon reflex, and HbA1c was investigated using multiple regression analysis. Besides the adjusted variables, BMI and HbA1c were found to be significant independent variables (Table 4).

**Table 4:** ba-PWV adjusted for age and blood pressure and relationship with each parameter. R² = 0.79, adjusted R² = 0.55, VIF < 1.95, * p < 0.05, ** p < 0.001. β: standardized partial regression coefficient; CI: confidence interval; VIF: variance inflation factor; SBP: systolic blood pressure; BMI: body mass index; HbA1c; hemoglobin A1c; ba-PWV: brachial-ankle pulse wave velocity.

	β	95% CI		p-value	VIF
		Lower limit	Upper limit		
Age	0.206	0.824	11.650	<0.05*	1.931
Sex	0.073	-67.191	176.721	0.375	1.597
SBP	0.442	6.567	12.781	<0.001**	1.214
BMI	-0.327	-41.696	-13.516	<0.001**	1.683
Knee joint extensor strength	-0.150	-28.731	2.601	0.101	1.953
Number of years of disease	0.068	-2.740	7.979	0.335	1.166
Achilles tendon reflex negative	0.113	-14.168	179.734	0.093	1.063
HbA1c	0.142	2.764	138.319	<0.05*	1.123

Binomial logistic regression analysis of ba-PWV divided into two groups by arterial stiffness index, ≥ (or <) 1,400 cm/s, showed that SBP (OR = 1.11, 95% CI = 1.05-1.18, p < 0.001), BMI (OR = 0.81, 96% CI = 0.688-0.95, p < 0.01), and number of years with disease (OR = 1.16, 98% CI = 1.05-1.27, p < 0.001) were found to be significant independent variables (Table 5).

**Table 5:** Relationship between arterial stiffness and each parameter adjusted for age and blood pressure. * p < 0.05, ** p < 0.01, *** p < 0.001. CI: confidence interval; SBP: systolic blood pressure; BMI: body mass index; HbA1c: hemoglobin A1c.

	Odds ratio	95% CI		p-value
		Lower limit	Upper limit	
Age	1.05	0.99	1.12	0.11
Sex	1.04	0.24	4.50	0.96
SBP	1.11	1.05	1.18	<0.001***
BMI	0.81	0.69	0.95	<0.01*
Knee joint extensor strength	0.83	0.67	1.04	0.10
Number of years of disease	1.16	1.05	1.27	<0.001**
Achilles tendon reflex negative	0.30	0.08	1.11	0.07
HbA1c	1.69	0.70	4.11	0.24

**Figure 2:**
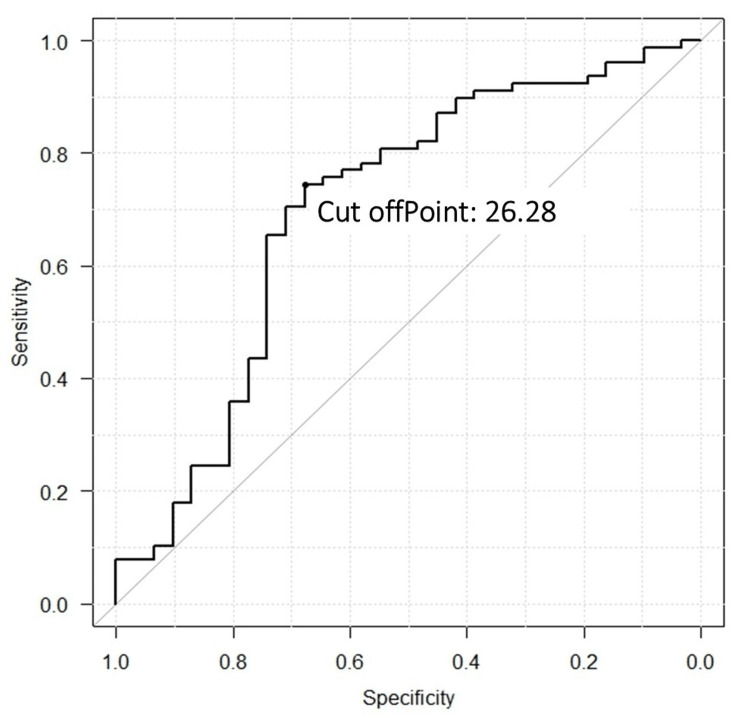
Cut-off value of BMI at which ba-PWV is >1,400 cm/s. The cut-off value for BMI with ba-PWV > 1400 cm/s was 26.28 kg/m². BMI: body mass index; ba-PWV: brachial-ankle pulse wave velocity.

The cut-off BMI value at which ba-PWV was >1,400 cm/s was calculated to be 26.28 kg/m² (area under the curve = 0.70, 95% CI = 0.58-0.82) (Figure 2). Further, the positive test rate was 0.72 (95% CI = 0.62-0.80), the true prevalence was 0.62 (95% CI = 0.53-0.72), the sensitivity was 0.85 (95% CI = 0.75-0.93), the specificity was 0.51 (95% CI = 0.35-0.67), the positive predictive value was 0.74 (95% CI = 0.63-0.84), the negative predictive value was 0.68 (95% CI = 0.49-0.83), the diagnostic accuracy was 0.73 (95% CI = 0.63-0.81), the positive likelihood ratio was 1.75 (95% CI = 1.26-2.43), and the negative likelihood ratio was 0.29 (95% CI = 0.15-0.55).

**Figure 3:**
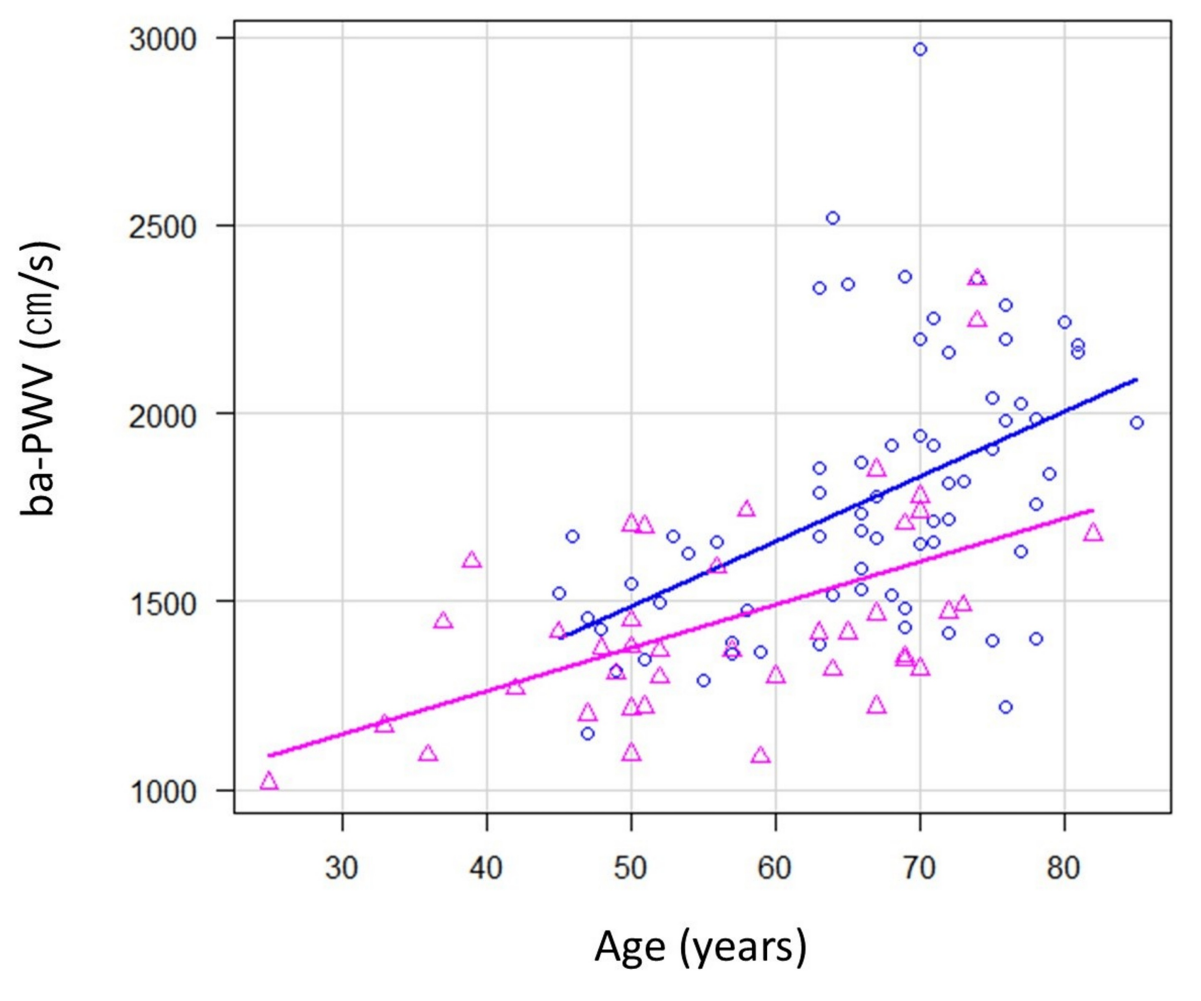
Relationship between ba-PWV and BMI considering the effect of aging. Factor (BMI cut-off value); △: above cutoff value; 〇: below cutoff value. Interaction between group variables and covariates (p = 0.25). BMI (F = 8.33, p < 0.01). Age (F = 32.34, p < 0.001). BMI: body mass index; ba-PWV: brachial-ankle pulse wave velocity; F: F-statistic.

In the relationship between ba-PWV and BMI with the effect of aging using ANCOVA, considering the effect of aging showed no interaction between the independent variables and covariates (p = 0.25), BMI (F = 8.33, p < 0.01) and age (F = 32.34, p < 0.001) were significant explanatory factors for ba-PWV (Figure 3).

## Discussion

This study investigated the relationship between arterial stiffness and body composition in patients with T2DM. Scatter plots showing the relationship between ba-PWV and BMI (Figure 2) and multivariate analysis revealed that an increase in ba-PWV was associated with a decrease in BMI and that the frequency of arterial stiffness increased when BMI was <26.28 kg/m2. The frequency of atherosclerosis increases when BMI falls below 26.28 kg/m². Although aging factors may also have influenced the decline in BMI and progression of atherosclerosis [20], there was no significant difference in age between the two groups divided by whether ba-PWV was above the age-specific reference value. Furthermore, the ANCOVA results showed no interaction between age and BMI; the regression line of the distribution chart was higher in the low BMI group than in the high BMI group, suggesting that BMI is a factor that influences ba-PWV, even with the effect of aging.

In a report on BMI in patients with T2DM, first-degree obesity (BMI = 25-29.9 kg/m²) was associated with a lower risk of death than normal weight (BMI = 18.5-25 kg/m²), whereas second-degree obesity (30-35 kg/m²) had a similar risk of death as normal weight (BMI = 18.5-25 kg/m²). In addition, the worst life prognosis [21] was reported for patients who were underweight (BMI < 18.5 kg/m²). While the risk of mortality is also high among patients with fourth-degree obesity (BMI > 40 kg/m²) in a previous study [22], the participants in this study had BMIs ranging from 17.82 to 37.34 kg/m². Thus, participants with a BMI of >40 kg/m² were not included. The ideal BMI for patients with T2DM would be between 25 and 30 kg/m², with the lowest risk of mortality, and the cut-off values for BMI indexed to atherosclerosis detected in this study were comparable to those in terms of mortality risk. However, it is recommended to maintain a body weight within an appropriate range to prevent the new onset of arteriosclerotic diseases and diabetes [23,24]. Further, prevention of diabetes onset is different from weight control after onset.

In chronic diseases, weight loss is a factor that worsens life expectancy, and the existence of the “obesity paradox” [25-28] has been reported. According to the obesity paradox, being overweight in older adults may function as a reserve (metabolic reserve) against sarcopenia, frailty, osteoporosis, and nutritional insufficiency and may contribute to improved life outcomes [21]. In T2DM cases, disease progression is also thought to increase insulin resistance, leading to decreased muscle protein synthesis and skeletal muscle mass [29]. Hyperinsulinemia associated with elevated blood glucose levels and increased insulin resistance in T2DM is believed to induce vascular endothelial dysfunction. This elevated blood glucose level increases glucose uptake into endothelial cells via glucose transporter type 1, which in turn increases polyol pathway activity, protein kinase C pathway activity, and advanced glycation end-product formation and induces endothelial cell dysfunction. Furthermore, insulin resistance causes impaired signaling through these pathways, leading to progressive vascular endothelial dysfunction [30].

Therefore, the increase in insulin resistance with aging and disease progression induces a decrease in skeletal muscle mass, manifesting as a decrease in BMI. In contrast, we hypothesized that the metabolic reserve function is activated in participants with a certain degree of high BMI; meanwhile, in those without such a reserve, skeletal muscle mass is reduced, and insulin resistance is increased, resulting in impairment of vascular endothelial function detected as an increase in ba-PWV.

Limitations

The results of this study were presented in a cross-sectional survey, and it is impossible to conclude at this time that a decrease in BMI changes the risk of atherosclerosis, since a clear causal relationship must be demonstrated by longitudinal studies to determine that a decrease in BMI causes atherosclerosis. Regarding whether BMI can be used to screen for arterial stiffness, the positive likelihood ratio was 1.74, and the negative likelihood ratio was 0.29, indicating that BMI alone cannot be used to evaluate positive and negative arterial stiffness. In addition, this study did not collect information on medication status. Therefore, the analysis did not consider the effects of medication. In addition, information on low-density lipoprotein cholesterol has not been collected sufficiently, and studies that consider the effects of low-density lipoprotein cholesterol have not been conducted. Furthermore, this study examined how increased insulin resistance exacerbates atherosclerosis, although it is thought that increased visceral fat mass may also increase insulin resistance. We were not able to examine the difference between the effects of skeletal muscle loss on ba-PWV and visceral fat mass increase.

## Conclusions

This study found that BMI is associated with the development of atherosclerosis in patients with T2DM. BMI below 26.28 kg/m² was associated with an increased risk of atherosclerosis. This led to the proposal of a cutoff value for BMI in the assessment of arterial stiffness in T2DM patients. This cutoff value may be particularly useful in small medical facilities and home care where it is difficult to bring in specialized equipment. Future research should include longitudinal studies to clarify the causal relationship between BMI and atherosclerosis.

This report shows a negative correlation between arterial stiffness and BMI in patients with T2DM. On the other hand, it only explained the association between BMI and arterial stiffness and did not provide recommendations for behavioral guidelines for patients. In the future, it will be necessary to propose behavioral guidelines that encompass diet, exercise, and medication status as indicators for maintaining an appropriate BMI in patients with T2DM.
